# Modeling antecedent factors involved in behavioral intention towards technology application of genetically modified crops

**DOI:** 10.1080/21645698.2022.2057160

**Published:** 2022-04-05

**Authors:** Yahya Safi Sis, Amirreza Rezaei, Hamid Karimi, Pouria Ataei

**Affiliations:** a Department of Agricultural Extension and Education, College of Agriculture & Natural Resources, University of Tehran, Tehran, Iran; b Department of Agricultural Extension and Education, Faculty of Agriculture, University of Zabol, Zabol, Iran; c Department of Agricultural Extension and Education, Faculty of Agriculture, Tarbiat Modares University, Tehran, Iran

**Keywords:** Behavioral intention, ethical concerns, genetically modified crops (GMCs), risks, social impact

## Abstract

This research aimed to study behavioral intention toward genetically modified crop (GMC) technology. The statistical population was composed of all staff experts of Jihad-e Agriculture Organization of Iran (N = 837). The sample size was 310 agricultural experts taken by simple randomization. The data collection tool was a questionnaire. Data were analyzed by structural equations modeling. The results provided enough evidence to confirm the negative and significant effect of ethical concerns on behavioral intention toward GMC technology and the positive and significant effect of attitude toward technology and social impact on behavioral intention. According to the results, it is necessary to hold training courses inside and outside the country, adopt bottom-up management, use experienced and prospective managers, involve experts in planning and development of GMCs to a greater extent, and share personal experiences in training courses to change people’ attitude.

## 1. Introduction

The population on the globe is on the rise with an annual reported rate of 83 million individuals, so it is expected to approach 8.5 billion by 2030 and 9.7 billion by 2050.^1^ According to existing estimates, about 1 billion people are experiencing hunger despite the ever-increasing population rise and reduction of available farmlands.^2^ Most crop varieties are on the verge of their genetic potential and it is hard to believe that varieties may be achieved with greater crop productivity. Consequently, a different technological revolution (following the Green Revolution) is needed in crop yields to feed the present population and maintain the existing food security level.^3–8^

As stated by Thomas Malthus, ethical justification on the use of biotechnology and genetic engineering is plausible through resorting to the fact that there is a need for feeding 10 billion people in the forthcoming years.^9^ Transgenic technology has witnessed rapid development with the scientific discovery of possible transfer of DNA from one organism to another. In the United States, the tobacco crop was initially manipulated transgenically for resistance to antibiotics.^10^ Afterward, transgenic foodstuffs have gained ground as an alternative approach to malnourishment and famine worldwide such that the farmlands of transgenic crops have amplified by 100-fold during the last 20 years, increasing from 1.7 million ha in 1996 to 179.7 million ha by the end of 2015. Recently, humans have adopted this technology as the most rapidly developing technical expertise.^11^ As defined by the World Health Organization (WHO), genetically modified (GM) foods are referred to those resulting from genetic engineering from organisms whose genetic material is subjected to such a modification that it will not occur in nature through reproduction and/or naturally recombinant material. GMOs are typically obtained by the introduction of a foreign gene to the genome or cell of an organism by the use of a virus, a gene gun, or direct injection into the nucleus.^12^

The risk management of the production of genetically modified crops (GMCs) relies heavily on the compliance of agricultural specialists with specific rules. Such compliance may vary depending on the attitudes and behavior of individual agricultural specialists. However, most scholars note that incomparably more research has been devoted to understanding consumers’ views on GM food in contrast to exploring the perceptions of agricultural specialists. Rather than attempting to gain an understanding of agricultural specialists’ attitudes and behavioral intention, the literature has predominantly focused on the experience of the adopters of GMCs and the interest of farmers in growing potentially permitted GMCs. specialists can properly impact the outlooks of customers, outrider farmers, and non-governmental organizations (NGOs).^13^ Accordingly, it is noteworthy that specialists function as doorkeepers capable of facilitating or impeding the publicizing and disseminating novelties throughout society.^14^ Hence, their outlooks toward a novelty will decisively contribute to motivating or hindering its expansion. Despite the noticeable advantages of GMC technology, most of its accomplishments remain to be accepted and are undervalued in the agricultural sector.^15^ In Iran, the annual import of GM products amounts to 5 billion USD, and transgenic foods account for 70% of imported products. To achieve independence, a strategy is to proceed toward the marketable production of domestic GMCs.

Advanced biotechnology is a potential armament in the fight against famine, thereby playing a role in resolving agricultural challenges including low revenues, insect invasion, plant extermination, and even drought.^2^ The technology of GMCs has enabled environmental protection, better food security, economic development, and human wellbeing. It has also provided enhanced revenues, improved environmental tension endurance such as drought, chilling, diseases, and pests, addressing the need for using insecticides damaging the environment being hazardous to human wellbeing, improving food quality, and manufacturing new facilities that are suitable for human wellbeing and industrial products.^16–22^

Nonetheless, introducing GM foods into societies emerged apprehensions regarding their environmental and human health impacts, which was associated with arguments on people’s right to be aware of their consumed foods.^10^ In recent years, supporters and contenders of such foodstuffs have been involved in arguments and conjectures regarding the advantages and disadvantages for the development of fundaments that are utilized for producing these products in terms of ideology, economics, politics, and the environment, but they have not reached a consistent agreement concerning the preparation of GM foods, which has led to the launch of the so-called ‘World War of Rhetoric.^18,23–25^ Mistrust, cynicism due to specialists’ lack of agreement, incoordination among risk managing entities, suboptimal communicating abilities regarding hazard and the history of dogmatic opinion, and misrepresentation and overstatement by supporter and contender parties have been other communication-linked challenges in arguments regarding biotechnology. Accordingly, no agreement and scientifically validated evidence can be found concerning the influences of using such foodstuffs in the academic associations.^1,26^

In Iran, the cynicism of administrators about such products has justified the lack of extensive marketable flourish of GMCs. Thus, all-inclusive information is unavailable regarding these crops. Besides, as users and farmers are uninformed about such products,^27^ they are not able to assess the possibilities and hazards imposed by such crops.^28,29^ As far as GMC technology is concerned, however, the separation between ‘real risk’ and ‘perceived risk,’ between ‘risk’ and ‘ethical concerns,’ or between ‘scientific’ concerns and ‘non-scientific’ concerns appears to be blurry. In such circumstances, the disputation about GMCs can apparently be orientated rationally via presenting proof of no confirmed complications and promoting technical information.^31^

The assertions of contenders versus producing and consuming GMCs can be itemized as the domination of producing seeds and the rest inputs of GMCs by special groups, worries about Iran’s dependency on GMCs (made by US and European corporations), and dependence of farmers on GM seeds and herbicides,^13,29^ the carcinogenicity of GMCs,^13,33^ possible development of novel viruses and toxins,^27,29,34^ possible development of allergies,^13,29,32–35^ and horizontal transfer of genes.^13,27,29,32,35,36^ To mitigate the above apprehensions concerning the probable hazards scientifically, it is necessary to consider the opinions of agricultural specialists on GMC technology.

Altogether, there is a necessity for providing users and farmers with scientifically validated information, encouraging Iran’s movement toward independence under present sanctions, and responding to objector’s apprehensions on the one hand, and there is no available investigation about the opinions of agricultural specialists on GMC technology on the other.^29^ This research, therefore, aims to address the question of ‘how knowledge, attitude, social and technical factors, and ethical concerns strengthening the behavioral intent agricultural specialists on GMC technology are interrelated to each other.’

## 2. Theoretical Framework and Research Hypotheses

A large number of the literature has been devoted to the attitude and behavior concerning the use of GMCs using several variables. A summary of the variables from the perspective of various investigators is presented in Table 1.

**Table 1.** The variables included in the research on the use of GMCs

Variables affecting behavioral intention toward GMCs	References
Knowledge of GMCs	Wunderlich and Gatto^37^; Alecsejeva^30^; Alecsejeva; Izumi et al.^39,40^; Usak et al.^41^; Bal et al.^42^; Amin et al.^43^
Perceived usefulness	Tsiboe et al.^44^; Vikan^45^; Immonen^46^; Ismail et al.^47,48^; Yao and Wang; Kagai^49^; Amin et al.^43^; Shehata and Cox^50^; Torres et al.^51^; Huang et al.^52^; Han (2006); Chen and Chen^53^; Springer et al.^54^; Baker and Burnham^55^
Perceived risk	Aleksejeva^38^; Ghasemi et al.^27^; Ghanian et al.^29^; Voss et al.^56^; Amin et al.^43^
Ethical concerns	Ataei et al.^57^; Ghoochani et al.^13^; Amin and Hashim^58^; Ormandy et al.^59^; Han (2006)
Attitude toward GMCs	Sorgo and Ambrožič-Dolinšek^60^; Ataei & Zamani^61^

In this research, a conceptual model was designed using Davis’s^62^ Technology Acceptance Model (TAM), which is among the most widespread and commonly applied theories in human behavior prediction. The Unified Theory of Acceptance and Use of Technology (UTAUT) was also considered here, which integrates eight authentic technology adoption models.^63^ In the end, the associations of variables were elucidated prior to the development of hypotheses and the conceptual model of the present research.

### 2.1. Ease of Use, Perceived Risk, and Perceived Usefulness

Based on investigations adopting the Theory of Planned Behavior (TPB), behavior is not formed merely by an individual’s behavioral inclinations and viewpoints, but the amassed capability for performing that behavior and his/her opinion about the ease of doing that behavior influence this procedure as well, which is certainly directed by a person’s discernment of the ease of applying a technology.^66^ *Ease of use* denotes the subjective probability shaped within an individual toward the facile employment of GMC technology for performing chores.^63^ Ease of use has had applications in the framework of the Decomposed Theory of Planned Behavior [DTPB;,^67^] TAM,^68^ and Technology Acceptance Model 2 [TAM 2; 69] as well as effort expectancy in UTAUT.^63^ The notion impacts perceived usefulness in TAM^68^ and TAM 2^69^ such that it increases ease of use of technology, thereby increasing one’s perceived practicality of that technology. This notion was redefined to perceived difficulty and its influence was examined differently.^70^

A person’s perception is a crucial determinant affecting the acceptance of GMC technology. Perceived practicality and perceived hazard are two critical notions having uppermost usage in research on GM parallel to the notions of knowledge and attitude (Table 1). Perceived practicality has had applications in DTPB,^67^ TAM,^68^ and TAM 2^69^ as well as performance expectancy in UTAUT.^63^ *Perceived usefulness* is used to denote the subjective probability grown inside an individual regarding an order of magnitude that accessible GM foods are valuable to feed society.^68^ *Perceived risk* highlights the subjective probability grown within a person regarding the environmental and human health hazards of eating accessible GM foods.^19,23,71^ Ghanian et al.^29^ drew a conclusion that agricultural specialists were informed about the environmental benefits and possible hazards of GM products, many of them accepted that GM foods could promote food security and expedite rural expansion, and the majority endorsed the use of labels on such foodstuffs. Their final opinion was that perceived advantages were positively correlated to perceived possible hazards of GM products. Amin and Hashim^58^ used the impact of perceived risk on perceived usefulness in their theoretical model. The theoretical models of many behavioral studies have highlighted the effect of perceived risk on the behavior of GMC technology usage [e.g.^13^, ^58^, ^70^] In TAM, Davis et al.^68^ have focused on the influence of perceived usefulness on attitude. The survey of Amin and Hashim^58^ demonstrated that people’s perception of multifaceted subjects (e.g. gene technology) should be regarded as a multi-sided procedure. They claimed that perceived usefulness to have more effectiveness than perceived risk and that perceived usefulness was the critical variable influencing attitude toward GMCs. Moreover, the authors described that perceived usefulness significantly influenced the attitude toward GM foodstuffs.

The following hypotheses are presented based on the above descriptions:

**H1**: ease of use has a significant influence on perceived usefulness,

**H2**: perceived risk has a significant influence on perceived usefulness,

**H3**: perceived risk has a significant influence on behavioral intentions toward GMC technology, and

**H4**: perceived usefulness has a significant influence on attitude toward GMCs.

### 2.2. Knowledge and Attitude

Indeed, scholars have often applied knowledge in the behavioral field of GM products exactly the same as attitude and perceived risk (Table 1), which suggests its effective role in making decisions on using GMC technology. *Knowledge* embraces the entire information concerning a given field that is saved in one’s longstanding memory and is occasionally serves as an element linking one’s value system to attitudes, being capable of affecting behavior.^27^ Besides, *attitude* denotes the extent that a person evaluates a given behavior, issue, or entity to be optimum or non-optimum.^72^ People’s range of knowledge can affect their behavioral intents toward GMC technology via attitude,^11,13,73^ and one’s perception of the risk of GMCs is also determined by the person’s knowledge.^13,34,74^ Therefore, three hypotheses are made as follows:

**H5**: knowledge of GMCs has a significant influence on perceived risk,

**H6**: knowledge of GMCs has a significant influence on attitude toward such crops, and

**H7**: attitude toward GMCs has a significant influence on behavioral intention toward such products.

### 2.3. Attitude Toward Technology and Environment

Attitude toward GMC technology has been shown to be influenced by environmental attitudes and technology attitudes, and these particular attitudes can either impact behavior via attitude or have a direct effect on behavioral intent toward GMCs.^74^ Individuals with a negative attitude to technology will have a negative attitude to GMCs as well.^75^ Besides, optimistic attitude to technology and upper education level are parameters affecting positive attitude toward GMCs in males to a higher degree than in females.^76–78^ The same as attitude to nature, it should also be considered that individuals seeking their own revenues most often recognize the hazards of GMCs, but those with greater environmental-friendly beliefs are inclined to identify the advantages of such crops.^79^ Based on the above, two hypotheses are considered as below:

**H8**: Attitude toward the environment has a significant effect on attitude toward GMCs.

**H9**: Attitude toward technology has a significant effect on behavioral intention toward GMCs.

### 2.4 Social Impact

Studies rooted in TPS [e.g. 64,65] mostly demonstrate that behavior is impacted by behavioral intents, which are in turn influenced by not only attitudes but also behaviors being expectable within the social environment and personal standards (social environment expectations).^66^ Social impact denotes the order of magnitude that a person’s decision is influenced by the opinions of other individuals, whether to adopt or refuse the system.^63^ This idea was found to be effective in behavioral intent in UTAUT.^63^ Kim^80^ presented evidence that the ecological concerns of GMC customers were associated with their negative social reactions to procuring these products. Thus, a hypothesis is formed denoting that

**H10**: social impact has a significant effect on behavioral intention toward GMC technology.

### 2.5. Ethical Concerns

A matter of public apprehension concerning the use of GMC technology is the value and ethical concerns referring to the subjective opinions of the public regarding the perfection or imperfection of a certain behavior.^14^ Ethical concerns have a negative impact on the behavior toward GMC technology usage.^13,58^ Such a perception originates from public views based on which genetic engineering disrupts nature^81^ and manipulates God’s work and creation.^82,83^ Therefore, a hypothesis is presented below

**H11**: ethical concerns have a significant effect on behavioral intentions toward GMC technology.

**Figure 1.**
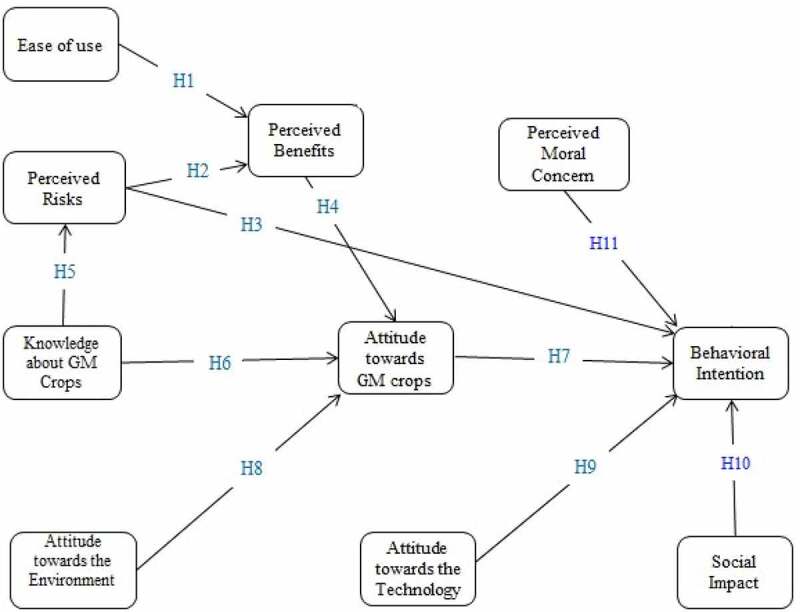
The conceptual model of the research.

Based on the above subsections, the conceptual model was designed here as illustrated in Figure. 1 (the study hypotheses are also introduced into the model). According to the conceptual framework, it can be stated that farmers’ knowledge of GMCs forms their attitude toward these crops and their perceived risk of their consumption. In other words, as experts gain more knowledge about GMCs, the risks will become clearer for them and their attitudes toward the use or nonuse of these crops will shape. On the other hand, the ease of GMC technology use can better show the advantages of GMCs to experts. When advantages are clearer, experts will gain positive attitudes toward them. But, if experts have ethical concerns over GMCs, their intention to use these crops will be influenced so that the process of deciding to use this technology may be challenged. Furthermore, society’s perspective on GMCs can influence experts’ behavioral intention to use or not to use them. In other words, if people have a positive perspective on GMCs and perceive that they are useful for society, experts’ intentions to use them will be strengthened.

## 3. Methods

The research was a descriptive correlational study carried out by the survey method. The statistical population was composed of staff experts of Jihad-e Agriculture Organization of Eastern Azerbaijan province (N = 837). The sample size was determined by Krejcie and Morgan’s table to be 264 for this statistical population, but it was increased to 310 people to reduce the error of the method of the structural equations. After the sample size was determined, a total of 400 questionnaires were sent to the participants to compensate for possibly unanswered questionnaires. Finally, 310 valid questionnaires were collected. According to the 10X rule, each hypothesis needs 10–20 samples. Given that the research tested 11 hypotheses and 310 questionnaires were collected, it can be said that this rule was complied with.^84^ Haire et al.^84^ suggest that the sample size should be equal to 10 times the number of independent variables in the most complex regression in the PLS path model (i.e., considering both measurement and structural models). The sample was taken by simple randomization. The main data collection instrument was a questionnaire composed of 11 sections for demographic-social characteristics, knowledge of GMC, attitude toward GMC, attitude toward technology, attitude toward the environment, perceived benefits, perceived risk, ease of use, social impact, ethical concerns, and behavioral intention toward GMC technology. The questionnaire was inspired at the design phase by the standard questionnaire of Davis^62^ and the literature review. The items were assessed on a five-point Likert scale ranging from ‘completely disagree’ to ‘completely agree’ (for items of knowledge of GMC, attitude toward GMC, attitude toward technology, attitude toward the environment, social impact, ease of use, ethical concerns, perceived benefits, and perceived risk) and from ‘very low’ to ‘very high’ (for items of behavioral intention toward GMC technology) (Table 2). The face and content validity of the questionnaire was verified by a panel of GMCs and behavioral experts. The reliability of the research instrument was estimated by Cronbach’s alpha in a pretest on 30 individuals of the respondents (randomly selected and not included in the main sample). The results are presented in Table 2. Since the alpha value was 0.70 or higher, the instrument was accurate enough to measure the variables. Cronbach’s alpha is a measure of internal consistency; that is, how closely a set of items are related as a group. It is considered to be a measure of scale reliability. Cronbach’s alpha tests to see if multiple-question Likert scale surveys are reliable. These questions measure latent variables – hidden or unobservable variables. Cronbach’s alpha will tell you how closely a set of test items are related as a group.^85^ Data were subjected to descriptive statistics (frequency, percentage, mean, and standard deviation) to describe the status of age, gender, educational level, work experience, and employment type and to inferential statistics (Pearson’s coefficient of correlation) to check their correlation using the SPSS_20_ software package. Also, they were modeled by structural equations in the AMOS_24_ software package to determine the causal relationships of the variables influencing behavioral intention toward GMC technology.

**Table 2.** The items included in the questionnaire and their alpha coefficient

Variable	Indicaors	Alpha
Perceived risks	The pollination of GMCs with conventional plants poses a risk to the environment.	0.78
	New viruses and toxins may be developed by GMCs.	
	GMCs are a threat to the genetic diversity of plants.	
	GMCs are harmful to plants and animals.	
	GMCs may be harmful to people who are allergic to a specific food.	
	Genetic mutation due to the consumption of GMCs is very harmful to people.	
Perceived benefits	GMCs are a solution to increasing food production.	0.75
	GMCs can contribute to environmental conservation.	
	GMCs can contribute to fighting plant pests and diseases.	
	GMCs can contribute to coping with drought.	
	GMCs have better taste and quality than conventional foodstuffs.	
	GMCs are effective in reducing the use of pesticides and the emission of greenhouse gases.	
Knowledge of GMCs	GMC technology does not differ from traditional plant breeding methods.	0.73
	Genetic modification means the transfer of a gene from one living organism to another.	
	Unlike organic products, GMCs are not subjected to safety assessment.	
	It is not possible to have a gene exchange between animals and plants.	
	Gene editing technology, CRISPER, is the same as GMC technology.	
Attitude toward GMCs	The planting of GMCs is reasonable.	0.82
	The release of GMCs into the environment has no problem.	
	It is OK to produce foodstuff by GMC technology.	
	It is OK to consume GMC.	
	The planting of GMC is for the benefit of the country.	
	Oppositions with GMC have no scientific logic.	
Attitude toward technology	Technology is necessary for progress and development.	0.84
	No technology is inherently harmful.	
	Technology application should be accompanied by planning and research about its ramifications.	
Attitude toward the environment	By manipulating nature, people trigger disruption in its natural trend.	0.80
	The balance of nature is robust enough to cope with the effects of modern technologies.	
	If the present conditions persist, a huge environmental disaster will happen.	
	‘Environmental crises’ that have been attributed to human actions have been exaggerated.	
	Humans should let the other living organisms survive too.	
Perceived ethical (Moral) concerns	Genetic modification is interference with the work of the Creation.	0.77
	Genetic modification is against religious rules.	
	Genetic modification is interference with nature.	
	Genetic mixing is ethically wrong.	
	GMCs are artificial products.	
East of use	The training of GMC technology to farmers will be an easy task.	0.72
	The extension of GMC technology will be an easy task.	
	Farmers will accrue more skill by more use of GMC technology.	
	There is no need for complicated agricultural machinery to use GMC technology.	
	It is easy to supply inputs and market GMCs.	
Social impact	Public people have a positive view on GMC technology.	0.84
	The managers of the agricultural sector think that the use of GMC technology is for the benefit of the agricultural sector.	
	My colleagues think that GMC technology should be in agriculture.	
	I think that the use of GMC technology in my job shows I am up-to-date.	
	My friends expect me to have enough experience and familiarity with GMC technology.	
Behavioral intention	I agree with the consumption of edible GM oils.	0.83
	I intend to consume GMCs (corn, soybean, and potato).	
	I have the willingness to use GM fruits.	
	I recommend the consumption of GM white and red meat (derived from GM livestock) to my friends and relatives.	
	I have the willingness to plan and attempt for the infrastructure to grow GMCs.	
	I have the willingness to use advanced biotechnology techniques and genetic engineering in the agricultural sector.	
	I have the willingness for the research, training, and extension of agronomic methods for GMCs.	
	I have the willingness to produce GMCs on a large scale.	

## 4. Results

### 4.1. Descriptive Statistics

Regarding the description of the demographic-professional characteristics of the statistical community, Table 3 shows that 80% of 310 respondents (i.e. 248 people) were male and 20% (i.e. 62 people) were female. In terms of age, about half of the participants were in the range of 36–50 years. The highest frequency of educational level (55%) was for the bachelor’s level. Most respondents had a job experience of 16–20 years (44%) and about two-thirds of them were permanently employed in the organization.

**Table 3.** Distribution of the participants with respect to their demographic characteristics

Variable	Level (stratum)	Percentage
Educational level	B.Sc.	55
	M.Sc.	31
	Ph.D.	14
Job experience	5 years or lower	9
	5–10 years	10
	11–15 years	23
	16–20 years	44
	21 years or higher	14
Age	18–35	21
	36–50	51
	51–76	28
Employment status	Permanent	73
	Temp-to-perm	27

### 4.2. The Measurement Model of the Research

The model addressed the Factors Involved in Behavioral Intention toward Technology Application of Genetically Modified Crops. First, we dealt with the fit of the measurement model including the validity and reliability of the research constructs. Hair et al.^84^ have proposed three metrics for the reliability of research constructs: (i) reliability of the individual items, (ii) composite reliability, and (iii) average variance extracted (AVE). The reliability of an individual item refers to the factor load of each observed variable. To determine the reliability of constructs, the modern measure of composite reliability (CR) is used in addition to the traditional measure of Cronbach’s alpha. The advantage of CR over Cronbach’s alpha is that it measures the reliability of constructs not on an absolute basis but on the basis of the correlation of the constructs. The CR value of over 0.7 for a certain construct means the good internal reliability of that construct, but the values of smaller than 0.6 reflect unreliability. The CR values were found to be greater than 0.7 for all constructs of the research model (Table 4).

**Table 4.** Reliability and Convergent Validity Measures

Variable	Indicators	Factor Loading	t-value	Average variance extracted (AVE)	Composite reliability(CR)
Perceived risks	n1	0.879	11.45	0.760	0.864
	n2	0.865	8.22		
	n3	-	delete		
	n4	-	delete		
	n5	-	delete		
	n6	-	delete		
Perceived benefits	b1	0.627	6.15	0.530	0.770
	b2	0.720	9.35		
	b3	0.824	16.15		
	b4	-	delete		
	b5	-	delete		
	b6	-	delete		
Knowledge of GMCs	k1	0.807	13.61	0.675	0.912
	k2	0.784	14.90		
	k3	0.844	15.79		
	k4	0.840	14.22		
	k5	0.830	12.58		
Attitude toward GMCs	at1	0.781	21.30		
	at2	0.762	21.21		
	at3	-	delete		
	at4	0.706	15.33		
	at5	0.645	10.58		
	at6	0.704	14.96		
Attitude toward technology	att1	0.834	37.93	0.775	0.932
	att2	0.904	60.23		
	att3	0.906	73.20		
Attitude toward the environment	a1	-	delete	0.665	0.798
	a2	-	delete		
	a3	0.775	8.405		
	a4	0.854	12.378		
	a5	-	delete		
Perceived ethical (Moral) concerns	e1	0.781	30.46	0.635	0.897
	e2	0.857	34.43		
	e3	0.779	22.42		
	e4	0.808	28.60		
	e5	0.755	20.57		
East of use	co1	0.780	3.24	0.612	0.759
	co2	-	delete		
	co3	-	delete		
	co4	0.784	3.24		
	co5	-	delete		
Social impact	s1	0.724	15.81	0.567	0.839
	s2	0.791	27.77		
	s3	0.782	21.33		
	s4	0.712	16.57		
	s5	-	delete		
Behavioral intention	I1	-	delete	0.609	0.861
	I2	-	delete		
	I3	-	delete		
	I4	0.829	43.37		
	I5	-	delete		
	I6	0.812	29.55		
	I7	0.795	26.10		
	I8	0.676	19.63		

After reliability, the second measure of the fit of measurement models is convergent validity. AVE has been introduced as a measure of convergent validity that examines the correlation of a construct with its own questions (indicators). AVE expresses the average variance shared by a construct and its own indicators. An AVE value of >0.5 suffices and it was more than 0.5 for the constructs of model. The validity of the research model was measured by the divergent validity measurement matrix using the Fornell-Larcker method. This measure of discriminant validity, i.e. divergent validity, is acceptable when the AVE value for each construct is greater than the variance shared between the construct and other constructs of the model (the square of the correlation coefficient between the constructs). To check it, the values of all correlations of the constructs are compared with the square root of AVE for each construct as shown in Table 4. If these values are higher than all correlations, the Fornell-Larcker criterion has been met. This has been satisfied in the present research and in the following matrix. According to Table 5, the Fornell-Larcker criterion was used in the context of diagnostic validity to check the significance of the contribution of indicators in accounting for at least 50% of the variance of the selected construct. The results revealed that the square root of AVE was greater than all existing correlations and the discriminant validity, which is a traditional measure of the precision of measurement models, was satisfied.

**Table 5.** The matrix of divergent (discriminant) validity measure by the Fornell-Larcker method

Variable	X1	X2	X3	X4	X5	X6	X7	X8	X9	X10
**X1**	0.880									
**X2**	0.030	0.815								
**X3**	0.022	−0.236	0.721							
**X4**	0.646	0.001	0.012	0.781						
**X5**	−0.043	−0.116	−0.017	0.041	0.782					
**X6**	−0.056	−0.122	0.208	−0.021	0.046	0.821				
**X7**	−0.001	−0.310	0.220	−0.009	0.121	0.055	0.728			
**X8**	−0.325	0.004	0.033	−0.495	0.034	0.052	0.058	0.797		
**X9**	−0.069	0.084	−0.073	−0.066	−0.126	−0.033	−0.147	−0.019	0.872	
**X10**	0.441	−0.017	−0.016	0.570	0.016	−0.013	0.032	−0.474	−0.094	0.753

**X1 =** Attitude toward Technology, **X2 = **Attitude about Environment, **X3 = **Attitude toward the GM Crops, **X4 = **Behavioral Intention, **X5 = **Ease of Use, **X6 = **Knowledge about GM Crops, **X7 =** Perceived Benefits, **X8 = **Perceived Ethical (Moral) Concern, **X9 = **Perceived Risks, **X10 = **Social Impact

Based on the results for Cronbach’s alpha (Table 2), CR, and AVE derived from the analyses and software output (Table 4), since the values are higher than the acceptable threshold for all variables, then the appropriateness of the model in terms of reliability and divergent and convergent validity is supported (Table 4).

### 4.3. Structural Model of Factors Influencing Behavioral Intention Toward GMC Technology

**Figure 2.**
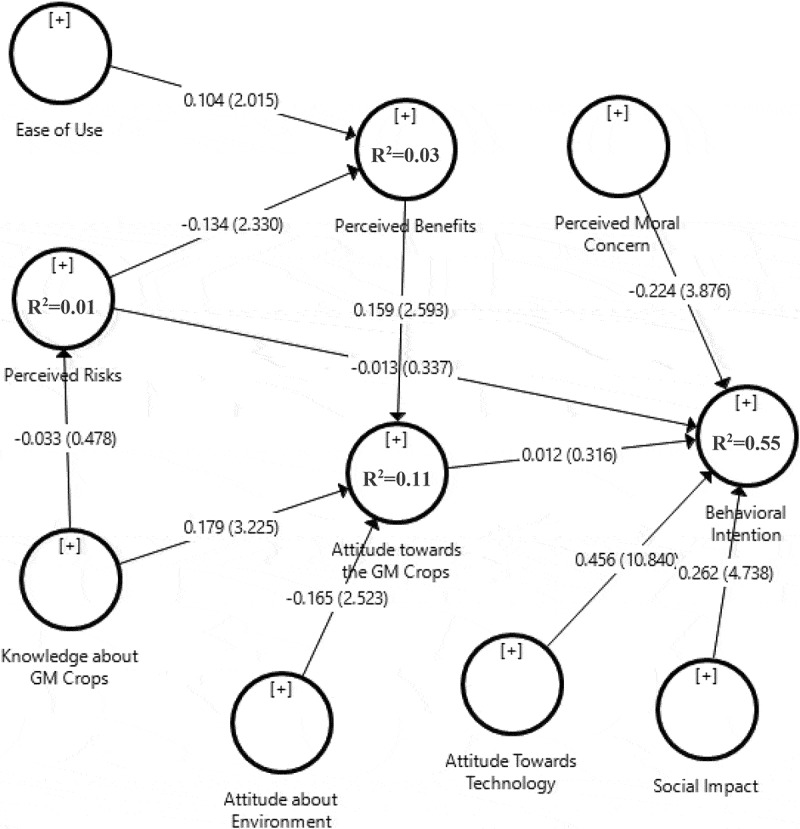
Path analysis of the research framework.

According to the data analysis algorithm by the PLS method, after fitting the measurement models, the fit of the structural model of the research is examined (Figure. 2). According to the structural model, the coefficient of determination (R^2^) of behavioral intention toward GMC technology indicates that 55% of its variance is accounted for by five constructs of ‘attitude towards GMCs,’ ‘attitude towards technology,’ ‘perceived risk,’ ‘social impact,’ and ‘perceived ethical concerns.’ The construct ‘Attitude towards the technology’ captured a great part of the variance of ‘behavioral intention’ (*p* < .01; β = 0.456). ‘Ethical concerns’ have a significant negative effect on ‘behavioral intentions towards GMC technology’ (*p* < .01; β = −0.224). and ‘Social impact’ has a significant positive impact on ‘behavioral intention’ (*p* < .01; β = 0.262). The variance of ‘attitude towards GMCs’ can also be captured by ‘knowledge of GMCs’ (*p* < .01; β = 0.179) and ‘attitude towards the environment’ (*p* < .01; β = −0.169).

In Table 6, the research hypotheses were tested according to standard coefficients and it was observed that out of 11 hypotheses, 8 hypotheses were confirmed. Among the eminent point in Table 6 is the significant effect of ‘attitude towards technology,’ ‘ethical concerns,’ and ‘social impact’ on ‘behavioral intentions towards GMC technology.’

**Table 6.** Structural parameter estimates and hypothesis results for the proposed model

Research hypothesis	Paths	Beta	T value	Conclusion
H1	Ease of use → perceived benefits	0.104	2.015^a^	Supported
H2	Perceived risks → perceived benefits	−0.134	2.330^a^	supported
H3	Perceived risks → behavioral intention	−0.013	0.337^ns^	Not supported
H4	Perceived benefits → attitude toward GMCs	0.159	2.593^aa^	Supported
H5	Knowledge about GMCs → perceived risks	−0.033	0.478^ns^	Not supported
H6	Knowledge about GMCs → attitude toward GMCs	0.179	3.225^aa^	Supported
H7	Attitude toward GMCs → behavioral intention	0.012	0.316^ns^	Not supported
H8	Attitude toward the environment → attitude toward GMCs	−0.165	2.523^aa^	Supported
H9	Attitude toward the technology → behavioral intention	0.456	10.840^aa^	Supported
H10	Social impact → behavioral intention	0.262	4.738^aa^	Supported
H11	Perceived ethical (moral) concerns → behavioral intention	−0.224	3.876^aa^	Supported

^a^Significant in 95% ** Significant in 99% ^ns^ Non Significan

## 5. Discussion and Conclusion

This research examined the attitude, knowledge, and behavioral parameters influencing the behavioral intention toward GMC technology among investigators of agricultural research centers according to TAM and a review of published studies. Our theoretical outcomes can be helpful in enriching the scientific work and can also be of practical use by strategists and organizers of agricultural genetic engineering.

The causal model analyzed here reveals that ‘social impact’ is the most influential variable in the prediction of ‘behavioral intention regarding GMC technology,’ which corresponds to Ghoochani et al.^13^ stating that social impact predicts behavioral intention to eat GM rice. It is speculated from this result that one’s companions and families can crucially affect his/her behavior toward the acceptance of GMC technology. It is recommended to establish brainstorm meetings and symposia on GMC technology continually to achieve a global agreement among investigators. In such meetings, attempts can be made to reinforce the morale of creativeness and invention among participants and to elucidate the possibilities and hazards of GMCs to hamper the nonscientific and disinterested impacts that individuals possibly attempt to exert on each other.

The results demonstrate a significant positive effect of knowledge about GMC technology attitude toward GMCs. Wunderlich and Gatto^37^ point out that it is essential to discriminate between acquaintance with GMOs and their scientific apprehension because people who have more knowledge of GMOs are more impervious to genetic engineering while there is a lower negative attitude toward GMOs in those at greater educational ranks. This suggests that there is a connection between technical knowledge, information resources, perceived risk, and attitude toward GMO products. Aleksejeva^30^ commented that specialists were deeply knowledgeable about genetic engineering technology leading to not having a fanatical attitude toward GMOs as had a mediocre consumer in the EU. A report by Ghasemi et al.^27^ indicates that people’s low knowledge had a negative impact on behavioral intention toward GMC technology and that GMC knowledge might be further developed if the systems for analyzing risks acted transparently.

As shown in Table 3, perceived usefulness has a positive effect on attitude toward GMCs, which is in line with that of Chen and Li^86^ who found positive impacts of people’s attitude on perceived usefulness and perceived usefulness on consumers’ attitude toward GMCs. Accordingly, it is proposed to accept a robust communication approach between investigators and specialists with the aim of informing the advantages of GMCs by investigators to specialists and by specialists to farmers specifically and the public generally. It can also be practical to establish a commission integrating three divisions of research, training, and extension. Besides, it is recommended to pave the route such that specialists can enjoy from preparation courses both nationally and internationally, bottom-up managing, using qualified and futuristic executives, further engagement of specialists in planning and developing GMCs, and governmental sponsorship as these can considerably have a contribution to promoting the attitude of specialists.

The findings unveiled a significant negative influence of ‘attitude toward the environment’ on ‘attitude towards GMCs.’ Ghoochani et al.^34^ consider that knowledge can positively impact the perceived usefulness of GMCs and that educators and executives have greater concerns about the safety of GMCs whereas farmers have higher environmental concerns. Apparently, environmental destruction by developing GMCs,^13,27,34,87^ its other facets including the horizontal transmission of genes,^13,27,29,35^ and the expansion of resilient weeds^13,29,34,35^ are challenges causing concerns in respondents as they have not received decisive responses from investigators. Nevertheless, considering the rising disputes of adversaries and proponents, this is accompanied by the impact of parochial groups and their advertisements (particularly among farmers, users, and less literate individuals) instead of emphasizing scientific resources and contents. In the attitude domain, the oratory facets and the skill of communicating and influencing the public’s thoughts have wrongly substituted the search for scientifically obtained proof. To address this challenge, it is suggested to organize sessions regularly and purposefully for adversaries and proponents who have expertise in various areas such as biotechnology, medical science, social science, economics, and agriculture in a scientific setting away from opinions directed politically, fractionally, and religiously. Publication of the conclusions from such gatherings can be helpful in avoiding the nonscientific and fanatical influences of both proponents and adversaries on the public. Additionally, GMC specialists are a validated information resource for farmers and users and have the responsibility of cooperating with other players to elucidate all the probable hazards stated above for such foodstuffs. Consequently, it is necessary for all actors involved in the areas of agriculture, health care, and medical science, and decision-makers of managing risk and communication procedures to participate in this process. It is suggested to focus on the attitude and behavior of specialists and investigators in forthcoming research.

It was detected that attitude toward technology had a marked contribution to behavioral intention toward GMCs. Despite the reality that all advanced and state-of-the-art technologies may experience some conflicts and disagreements to their development,^29^ it seems that individuals tending to adopt up-to-date technology show an inclination to adopt GMCs in spite of all objections concerning the products. As stated by Chen and Li,^86^ because gene technology is a novel technology, individuals can be reinforced through training to think positively about the technology and the environment. Grunert, Bredahl, Scholderer, (2003) believe that attitude toward GMCs is influenced by attitude toward the environment, attitude toward technology, and isolation from the market, which act profoundly and it is hard to change them through informing methods. Accordingly, subjective familiarity and clearly perceiving the advantages of GMCs are required to modify the attitudes. Therefore, people’s personal experiences are recommended to be shared in teaching sessions to alter their attitudes.

Additionally, moral concerns for GMCs were detected to have a significantly negative impact on behavioral intention. This corresponds to Ormandy et al.^59^ who stated a growing interest in GM animals in the past decade and the inclusion of subjective and social values in using these animals in this discipline. Apparently, this is related to religiously and culturally driven social objections persuaded based on the social-political background of society and administrative culture as ethical thoughts are contextualistic. Consequently, it is proposed that investigators focus on the impact of mental-social maturity on behavioral intention toward GMCs in the framework of such variables as age, education, and linking to social bodies in their forthcoming research.

## References

[1] Zhang M, Chen C, Hu W, Chen L, Zhan J. Influence of source credibility on consumer acceptance of genetically modified foods in China. Sustainability. 2016;8(9):899–921. doi:10.3390/su8090899.

[2] Delaney B. Safety assessment of foods from genetically modified crops in countries with developing economies. Food Chem Toxicol. 2015;86(23):132–43. doi:10.1016/j.fct.2015.10.001. 26456807

[3] Bloom V. Nourishing the planet in the 21st century. Plant science classroom material for high schools in Ontario. Ontario, Canada: Nutrients for Life Foundation; 2010.

[4] Ghadermarzi H, Ataei P, Karimi H, Norouzi A. The learning organisation approaches in the jihad-e agriculture organisation, Iran. Knowl Manag Res Pract. 2020;1–15. doi:10.1080/14778238.2020.1767520.

[5] Grunert KG, Bredahl L, Scholderer J. Four questions on European consumers’ attitudes toward the use of genetic modification in food production. Innovative Food Sci Emerg Technol. 2003;4(4):435–45. doi:10.1016/S1466-8564(03)00035-3.

[6] Sharma R (2012). Ensuring the success of feed the future: analysis and recommendations on gender integration. global agricultural development initiative issue briefs are published by the Chicago council on global affairs.

[7] Sorgo A, Ambrozis-Dolinsek J. The relationship among knowledge of attitudes toward and acceptance of genetically modified organisms (GMOs) among Slovenian teachers. Electron J Biotechnol. 2009;12(4):1–2. doi:10.2225/vol12-issue4-fulltext-1.

[8] Siegrist M. The influence of trust and perceptions of risks and benefits on the acceptance of gene technology. Risk Anal. 2000;20(2):195–204. doi:10.1111/0272-4332.202020. 10859780

[9] Devos Y, Maeseele P, Reheul D, Van Speybroeck L, De Waele D. Perceived moral concern in the societal debate on genetically modified organisms: a (re) quest for sense and sensibility. J Agric Environ per Moral Concern. 2008;21:29–61.

[10] Ma Y. Consumers’ different attitudes towards genetically modified food in the United States and China. Stud Asian Soc Sci. 2015;2(2):1–17. doi:10.5430/sass.v2n2p1.

[11] Abdullah AHM, Afrad MSI, Bhuiyan AAH, Haque ME, Islam T. Attitude and consumption of Bangladeshi professionals toward biotechnological products. Agric Food Sec. 2018;7(1):2–18. doi:10.1186/s40066-017-0155-z.

[12] Wong AY, Chan AW. Genetically modified foods in China and the United States: a primer of regulation and intellectual property protection. Food Sci Hum Wellness. 2016;5(18):124–40. doi:10.1016/j.fshw.2016.03.002.

[13] Ghoochani OM, Ghanian M, Baradaran M, Azadi H. Multi stakeholders’ attitudes toward Bt rice in Southwest, Iran: application of TPB and multi-attribute models. Integr Psychol Behav Sci. 2017;51(1):141–63. doi:10.1007/s12124-016-9358-2. 27498978

[14] Yazdanpanah M, Hayati D, Zamani GH. investigating agricultural professionals’intentions and behaviours towards water conservation: using a modified theory of planned behaviour. Inter J Environ Physiol Toxicol. 2011;9:1–22.

[15] Tonukari NJ, Omotor DG. Biotechnology and food security in developing countries. Biotechnol Mol Biol Rev. 2010;4:13–23.

[16] Al-Khayri JM, Hassan MI. Socio-Demographic factors influencing public perception of genetically modified. Am J Food Technol. 2012;7(3):101–12. doi:10.3923/ajft.2012.101.112.

[17] Ataei P, Ghadermarzi H, Karimi H, Norouzi A. The process of adopting entrepreneurial behaviour: evidence from agriculture students in Iran. Innov Educ Teach Int. 2021;58(3):340–50. doi:10.1080/14703297.2020.1734476.

[18] Herdt R. The state of food and agriculture, 2003–2004: agricultural biotechnology: meeting the needs of the poor? Agric Econ. 2005;32(1):109–10. doi:10.1111/j.0169-5150.2005.t01-7-00008.x.

[19] Kramkowska M, Grzelak T, Czyzewska K. Benefits and risks associated with genetically modified food products. Ann Agric Environ Med. 2013;20:413–19. 24069841

[20] Pandey A, Kamle M, Yadava L, Muthukumar M, Kumar P, Gupta V, Pandey B, Pandey BK. Genetically modified food: its uses, future prospects and safety assessments. Biotechnology. 2010;9(4):444–58. doi:10.3923/biotech.2010.444.458.

[21] Rodríguez-Entrena M, Salazar-Ordóñez M. Influence of scientific-technical literacy on consumers’ behavioural intentions regarding new food. Appetite. 2013;60:193–202. doi:10.1016/j.appet.2012.09.028. 23063609

[22] Rao NC. Biotechnology for second green revolution in Indian agriculture. Productivity. 2013;54:126–41.

[23] Lukošiutė I, Petrauskaitė-Senkevič L. Evaluation of Lithuanian consumers’attitudes to genetically modified food. J Agribus Rural Dev. 2017;1:103–11.

[24] Stone GD, Altieri MA, Pental D, Richards P, Suryanarayana M, Tripp R. Both sides now: fallacies in the genetic-modification wars, implications for developing countries, and anthropological perspectives. Curr Anthropol. 2002;43(4):611–30. doi:10.1086/341532.

[25] Yang T, Ames G, Berning J. Determinants of consumer attitudes and purchasing behaviors on genetically modified foods in Taiwan. J Food Distrib Res. 2015;46:30–36.

[26] Verhoog H (2007). Organic agriculture versus genetic engineering. NJAS 54-4.

[27] Ghasemi S, Karami E, Azadi H. Knowledge, attitudes and behavioral intentions of agricultural professionals toward genetically modified (GM) foods: a case study in Southwest Iran. Sci Eng per Moral Concern. 2013;19:1201–27. 10.1007/s11948-012-9383-6 22843033

[28] Earle TC, Cvetkovich G. Social trust: toward a cosmopolitan society. London: Praeger (Westport, Conn.). 1995. 0275948455.

[29] Ghanian M, Ghoochani OM, Kitterlin M, Jahangiry S, Zarafshani K, Van Passel S, Azadi H. Attitudes of agricultural experts toward genetically modified crops: a case study in Southwest Iran. Sci Eng per Moral Concern. 2016;22:509–24. 10.1007/s11948-015-9653-1 26045394

[30] Aleksejeva I. EU experts’ attitude towards use of GMO in food and feed and other industries. Procedia Soc Behav Sci. 2014;110:494–501. doi:10.1016/j.sbspro.2013.12.893.

[31] Cui K, Shoemaker SP. Public perception of genetically-modified (GM) food: a nationwide Chinese consumer study. NPJ Sci Food. 2018;2(10):34–51. doi:10.1038/s41538-018-0018-4. PMC6550219 31304260

[32] Martinez-Poveda A, Molla-Bauza MB, Campo Gomis FJD, Martinez-Carrasco ML. Consumer-perceived risk model for the introduction of genetically modified food in Spain. Food Policy. 2009;34(6):519–28. doi:10.1016/j.foodpol.2009.08.001.

[33] Rzymski P, Królczyk A. Attitudes toward genetically modified organisms in Poland: to GMO or not to GMO? Food Sec. 2016;8(2):689–97. doi:10.1007/s12571-016-0572-z.

[34] Ghoochani OM, Ghanian M, Baradaran M, Alimirzaei E, Azadi H. Behavioral intentions toward genetically modified crops in Southwest Iran: a multi-stakeholder analysis. Environ Dev Sustain. 2018;20(1):233–53. doi:10.1007/s10668-016-9879-3.

[35] Mohapatra AK, Priyadarshini D, Biswas A. Genetically modified food: knowledge and attitude of teachers and students. J Sci Educ Technol. 2010;19(5):489–97. doi:10.1007/s10956-010-9215-x.

[36] Aerni P. Assessing stakeholder attitudes to agricultural biotechnology in developing countries. Biotechnol Dev Monit. 2001;47:2–7.

[37] Wunderlich S, Gatto KA. Consumer perception of genetically modified organisms and sources of information. Adv Nutr. 2015;6(6):842–51. doi:10.3945/an.115.008870. 26567205 PMC4642419

[38] Aleksejeva I. comparative analysis of GMO risk perception gap between EU consumers and Latvian experts involved in GMO decision-making process. New Challenges Econ Bus Dev. 2013;7:91–109.

[39] Izumi S, Mori H, Kusaba S, Okada T, Murayama T, Yamamoto T (2011). Japanese attitudes toward genetic engineering. Paper presented at the Symposium on Liberal Arts and General Education (Kyoto University, Japan).

[40] Jöreskog KG, Sörbom D. LISREL 8: structural equation modeling with the SIMPLIS command language. United State America: Scientific Software International; 1993.

[41] Usak M, Erdogan M, Prokop P, Ozel M. High school and university students’ knowledge and attitudes regarding biotechnology. Biochem Mol Biol Educ. 2009;37(2):123–30. doi:10.1002/bmb.20267. 21567719

[42] Bal Ş, Samancı NK, Bozkurt O. University students’ knowledge and attitude about genetic engineering. Eurasia J Math Sci Technol Educ. 2007;3:119–26.

[43] Amin L, Jahi JM, Nor ARM (2007). Attitude towards genetically modified soybean amongst the Klang Valley stakeholders. Paper presented at 2nd Bangi World Conference on Environmental Management, Bangi, Malaysia, pp. 630–645. 13–14 September.

[44] Tsiboe F, Nalley LL, Dixon BL, Danforth D, Delwaide AC, Nayga RM. Ghanaian consumers’ attitudes toward cisgenic rice: are all genetically modified rice the same? Ghana J Dev Stud. 2017;14(1):1–18. doi:10.4314/gjds.v14i1.1.

[45] Vikan R. Consumer response to genetically modified salmon: a study on benefit importance in the adoption process. New Zealand: University of Otago; 2015. 34–51.

[46] Immonen A-M. Essays on emotional influences in consumer food choice: understanding emotional intricacies in consumers’ price vs. ethicality trade-off decisions, and perceptions of genetically modified food products. Finland: Doctoral Dissertations. Department of Economics and Management University of Helsinki Finland; 2015.

[47] Ismail K, Soehod K, Vivishna S, Khurram W, Jafri SKA, Ramily MKB. Genetically modified food and consumer purchase intentions: a study in Johor Bahru. Int J Bus Soc Sci. 2012;3:41–58.

[48] Yao Q, Wang L. Consumer purchase intention towards genetically-modified food: beneficial, price, socio-demographic and label determinants. Int J Trade Econ Financ. 2012;3(3):176. doi:10.7763/IJTEF.2012.V3.195.

[49] Kagai KK. Assessment of public perception, awareness and knowledge on genetically engineered food crops and their products in Trans-Nzoia County, Kenya. J Dev Sustainable Agric. 2011;6:164–80.

[50] Shehata S, Cox LJ (2007). Attitudes of Hawaii consumers toward genetically modified fruit. M. A. Thesis. University of Hawaii.

[51] Torres CS, Suva MM, Carpio LB, Dagli WB. Public understanding and perception of and attitude towards agricultural biotechnology in the Philippines. In: International service for the acquisition of agri-biotech applications, southeast asian regional center for graduate study and research in agriculture, and college of development communication. College (Laguna, Philippines): University of the Philippines Los Baños. 1–50; 2006.

[52] Huang J, Qiu H, Bai J, Pray C. Awareness, acceptance of and willingness to buy genetically modified foods in Urban China. Appetite. 2006;46(2):144–51. doi:10.1016/j.appet.2005.11.005. 16469414

[53] Chen H-Y, Chern WS (2002). Consumer acceptance of genetically modified foods. Paper presented at the Annual Meeting of the American Agricultural Economics Association, Long Beach CA.

[54] Springer A, Mattas K, Papastefanou G, Tsioumanis A. Comparing consumer attitudes towards genetically modified food in Europe. In: presentation at the X th EAAE congress exploring diversity in the European agri-Food. System Zaragoza (August Spain). 1–14; 2002.

[55] Baker GA, Burnham TA. Consumer response to genetically modified foods: market segment analysis and implications for producers and policymakers. J Agric Resour Econ. 2001;8:387–403.

[56] Voss AGAJ, Spiller A, Enneking U. Farmer acceptance of genetically modified seeds in Germany: results of a cluster analysis. Inter Food Agribus Manage Rev. 2009;12:61–80.

[57] Ataei P, Ghadermarzi H, Karimi H, Norouzi A. The barriers hindering the application of the value chain in the context of rural entrepreneurship. J Agric Educ Ext. 2020;26(4):365–82. doi:10.1080/1389224X.2020.1726780.

[58] Amin L, Hashim H. Factors influencing stakeholders attitudes toward genetically modified aedes mosquito. Sci Eng per Moral Concern. 2015;21:655–81. 10.1007/s11948-014-9557-5 24906652

[59] Ormandy EH, Schuppli CA, Weary DM. Worldwide trends in the use of animals in research: the contribution of genetically-modified animal models. Altern Lab Anim: ATLA. 2009;37(1):63–68. doi:10.1177/0261192\90903700109. 19292576

[60] Sorgo A, Ambrožič-Dolinšek J. The relationship among knowledge of attitudes toward and acceptance of genetically modified organisms (GMOs) among Slovenian teachers. Electron J Biotechnol. 2009;12:1–2.

[61] Ataei P, Zamani N. Determinants of the transfer of sustainability learning in agriculture sector of Iran. J Agric Sci Technol. 2015;17:1437–47.

[62] Davis FD. Perceived usefulness, perceived ease of use, and user acceptance of information technology. MIS Q. 1989;7(9):319–40. doi:10.2307/249008.

[63] Venkatesh V, Morris MG, Davis GB, Davis FD. User acceptance of information technology: toward a unified view. MIS Q. 2003;27(3):425–78. doi:10.2307/30036540.

[64] Fishbein M, Ajzen I. Theory-based behavior change interventions: comments on Hobbis and Sutton. J Health Psychol. 2004;10(1):27–31. doi:10.1177/1359105305048552. 15576497

[65] Gardner GE, Troelstrup A. Students’ attitudes toward gene technology: deconstructing a construct. J Sci Educ Technol. 2015;24(2015):519–31. doi:10.1007/s10956-014-9542-4.

[66] Ajzen I. The theory of planned behaviour: reactions and reflections. Health Psychol Rev. 2011;5:97–144. 10.1080/08870446.2011.613995 21929476

[67] Taylor S, Todd PA. Understanding information technology usage: a test of competing models. Inf Syst Res. 1995;6(2):144–76. doi:10.1287/isre.6.2.144.

[68] Davis FD, Bagozzi RP, Warshaw PR. User acceptance of computer technology: a comparison of two theoretical models. Manage Sci. 1989;35(8):982–1003. doi:10.1287/mnsc.35.8.982.

[69] Mathieson K. Predicting user intentions: comparing the technology acceptance model with the theory of planned behavior. Inf Syst Res. 1991;2(3):173–91. doi:10.1287/isre.2.3.173.

[70] Bredahl L, Grunert KG, Frewer LJ. Consumer attitudes and decision-making with regard to genetically engineered food products–a review of the literature and a presentation of models for future research. J Consum Policy. 1998;21(3):251–77. doi:10.1023/A:1006940724167.

[71] Paarlberg R. GMO foods and crops: africa’s choice. N Biotechnol. 2010;27(5):609–13. doi:10.1016/j.nbt.2010.07.005. 20637906

[72] Ajzen I. The theory of planned behavior. Organ Behav Hum Decis Process. 1991;50(2):179–211. doi:10.1016/0749-5978(91)90020-T.

[73] Han JH. The effects of perceptions on consumer acceptance of genetically modified (GM) foods. USA: Doctoral Dissertations. Louisiana State University and Agricultural and Mechanical College; 2006.

[74] Bredahl L. Determinants of consumer attitudes and purchase intentions with regard to genetically modified food–results of a cross-national survey. J Consum Policy. 2001;24(1):23–61. doi:10.1023/A:1010950406128.

[75] Amin L, Md Jahi J, Md Nor A, Osman M, Mahadi N. Relationship between general attitude towards nature religion, custom, science and technological progress and attitude towards modern biotechnology. Malaysian J Environ Manage. 2005;6:73–86.

[76] Anunda HN, Njoka F, Shauri S, Halimu S. Assessment of Kenyan public perception on genetic engineering of food crops and their products. Bio Science. 2010;33:2027–36.

[77] Hoban TJ. Public perception and understanding of agricultural biotechnology. Cereals Foods World. 2004;43:21–22.

[78] Huijts NMA, Molin EJE, Steg L. Psychological factors influencing sustainable energy technology acceptance: a review-based comprehensive framework. Renew Sust Energ Rev. 2012;16(1):525–31. doi:10.1016/j.rser.2011.08.018.

[79] Amin L, Jahi M, J M, Nor A, Osman M, Mahadi N. Uncovering factors influencing Malaysian public attitude towards modern biotechnology. Asia Pac J Mol Biol Biotechnol. 2006;14:33–39.

[80] Kim YG. Ecological concerns about Genetically Modified (GM) food consumption using the Theory of Planned Behavior (TPB). Procedia Soc Behav Sci. 2014;159(13):677–81. doi:10.1016/j.sbspro.2014.12.467.

[81] Sjoberg L. Principles of risk perception applied to gene technology: to overcome the resistance to applications of biotechnology, research on risk perception must take a closer look at the public’s reasons for rejecting this technology. EMBO Rep. 2004;5(1S):S47–S51. doi:10.1038/sj.embor.7400258. 15459735 PMC1299214

[82] Gott M, Monamy V. Perceived moral concern and transgenesis: toward a policy framework incorporating intrinsic objections and societal perceptions. Altern Lab Anim: ATLA. 2004;32(1_suppl):391–96. doi:10.1177/026119290403201s64. 23577492

[83] Grunert KG, Bredahl L, Scholderer J. Four questions on European consumers’ attitudes toward the use of genetic modification in food production. Innov Food Sci Emerg Technol. 2003;4:435–45.

[84] Hair JF, Hult GTM, Ringle CM, Sarstedt M. A Primer on Partial Least Squares Structural Equation Modeling (PLS-SEM). 3rd. Thousand Oaks (CA): Sage; 2021.

[85] Tavakol M, Dennick R. Making sense of cronbach’s alpha. Inter J Med Educ. 2011;2:53–55. doi:10.5116/ijme.4dfb.8dfd. PMC4205511 28029643

[86] Chen MF, Li HL. The consumer’s attitude toward genetically modified foods in Taiwan. Food Qual Prefer. 2007;18(4):662–74. doi:10.1016/j.foodqual.2006.10.002.

[87] Zhong F, Marchant MA, Ding Y, Lu K. GM foods: a Nanjing case study of Chinese consumers’ awareness and potential attitudes. Agrobiotechnol Manag Econ. 2002;5:136–44.

